# Does the use of a closed-suction drain reduce the effectiveness of an antibiotic-loaded spacer in two-stage exchange Arthroplasty for Periprosthetic hip infection? A prospective, randomized, controlled study

**DOI:** 10.1186/s12891-019-2974-5

**Published:** 2019-12-04

**Authors:** Chi Xu, Cheng-Qi Jia, Feng-Chih Kuo, Wei Chai, Ming-Hua Zhang, Ji-Ying Chen

**Affiliations:** 1 Department of Orthopaedic Surgery, General Hospital of Peoples Liberation Army, No. 28 Fuxing Road, Beijing, 100853 Haidian District China; 2 Department of Orthopaedic Surgery, Kaohsiung Chang Gung Memorial Hospital and Chang Gung University, College of Medicine, Kaohsiung, Taiwan; 3 Department of Clinical Pharmacy Laboratory, General Hospital of Peoples Liberation Army, No. 28 Fuxing Road, Beijing, 100853 Haidian District China

**Keywords:** Closed-suction drain, Periprosthetic joint infection, Two-staged exchange arthroplasty, Antibiotic concentration, Antibiotic-loaded cement spacer

## Abstract

**Background:**

There is a concern regarding the use of a closed-suction drain (CSD) in two-stage exchange arthroplasty for periprosthetic joint infection as it may decrease the antibiotic concentrations in the joint fluids. The purpose of this study was to identify whether the use of a CSD could reduce local antibiotic concentrations following spacer implantation.

**Methods:**

A prospective, randomized, controlled trial was conducted at our institution between January 2018 and November 2018. We enrolled 32 patients undergoing two-stage exchange arthroplasty for periprosthetic hip infection with an interim cement spacer containing 4-g vancomycin and 2-g meropenem per 40-g methyl-methacrylate cement polymer. Patients were randomized and evenly divided into the study group (non-CSD) and control group (CSD group) by sealed envelopes. Drainage samples of joint fluids (*n* = 160) were collected every 24 h for the first five days following spacer implantation. The antibiotic concentrations of drainage samples were measured by high-performance liquid chromatography, and the bioactivities of the drainage samples against methicillin-sensitive and methicillin-resistant *Staphylococcus aureus* (MSSA and MRSA) and *E. coli* were assessed.

**Results:**

There was no significant difference in the decrease of vancomycin (study group vs. control group: 163.20 ± 77.05 vs. 162.39 ± 36.31; *p* = 0.917) and meropenem concentration (123.78 ± 21.04 vs. 117.27 ± 19.38; *P* = 0.548) between the two groups during the first five days following spacer implantation. All joint drainage samples in each group exhibited antibacterial activity against MSSA, MRSA and *E. coli*.

**Conclusions:**

The use of CSD following the implantation of an antibiotic-loaded cement spacer does not reduce the effectiveness of such a spacer in two-stage exchange arthroplasty.

(Chinese Clinical Trial Registry, ChiCTR-INR-17014162. Registered 26 December 2017.)

## Background

Periprosthetic joint infection (PJI) is one of the most disastrous complications following total hip arthroplasty (THA) and remains a challenging condition to treat. Two-stage exchange arthroplasty with the implantation of an antibiotic-loaded cement spacer (ACS) and reimplantation of a new prosthesis remains the widely held gold-standard treatment of chronic PJI [1]. The use of a cement spacer is based on the principle that the spacer releases the antibiotics gradually to keep local antibiotic concentrations high. However, the concentration of local antibiotics released from the ACS gradually decreases over time [2]. Previous studies have shown that organisms are able to grow and form a biofilm on cement spacers in a manner similar to that occurring on a metal component [3, 4]. Moreover, the organism on the spacer is likely to be tolerant to antibiotics loaded in the cement spacer, [5] which may increase the risk of treatment failure. Therefore, it is critical to identify the related factors that influence the effectiveness of the antibiotic-loaded spacer.

Although several randomized, controlled studies have suggested no inherent benefit with the use of closed-suction drainage (CSD) in primary total joint arthroplasty (TJA), [6, 7] it is still commonly used in revision TJA. This is because more complex procedures and prolonged operative time in revision procedures may result in substantial blood loss and a high risk for postoperative hematoma when compared with primary THA [8, 9]. Currently, there is a concern regarding the use of CSD in two-stage exchange arthroplasty and its effect on local antibiotic concentrations from ACS. The CSD drains the joint fluids containing the antibiotics released by the ACS, which may decrease the antibiotic concentrations in the joint fluids. This issue was also raised and debated in the 2018 International Consensus Meeting (ICM) [10]. However, it remains controversial as there has been no data that assessed the impact of the use of CSD following the implantation of a cement spacer in two-stage exchange arthroplasty.

Therefore, we conducted a prospective, randomized, controlled study 1) to evaluate whether the use of CSD could reduce the local antibiotic concentrations following spacer insertion and 2) to identify the association between drainage volume and antibiotic concentrations in the joint drainage.

## Methods

### Participants and randomization

This prospective, randomized, controlled study was registered in the public Clinical Trial Registry (ChiCTR-INR-17014162) and approval was obtained from the Clinical Trials and Biomedical Ethics Committee of our institution (S2017–085-02). The present study adhered to CONSORT guidelines. All participants were enrolled after written informed consent was obtained before randomization.

**Fig. 1:**
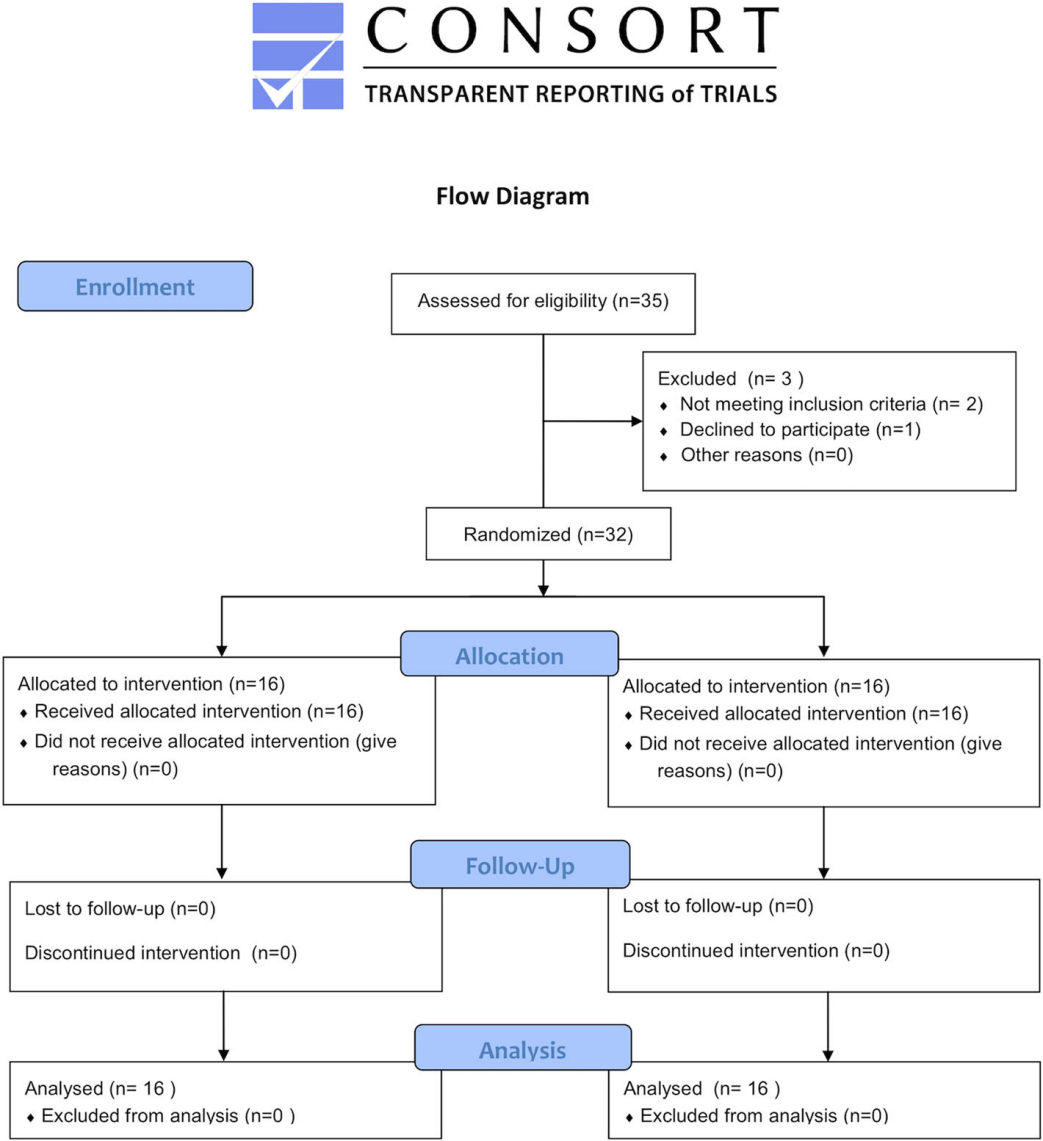
Study flowchart

Patients with chronic PJI of the hips and scheduled for two-stage exchange arthroplasty were included. PJI diagnosis was made using the Musculoskeletal Infection Society (MSIS) criteria [11]. Patients were excluded from the study if they had a previous allergic history to the listed antibiotics (vancomycin, meropenem, ceftriaxone, linezolid, or bone cement), preoperative hepatic or renal dysfunction, a malignant tumor, ongoing immunosuppressive agents, refusal to participate this study or participation in another clinical study. From January 2018 and November 2018, 35 patients with chronic PJI following primary THA were eligible for study enrollment. Three patients were excluded because of ineligibility. The remaining 32 patients were randomly assigned by means of a computer-generated randomization method to either the study group (non-CSD group, 16 patients) or the control group (CSD group, 16 patients) (Fig. 1). The study population included 22 women and 10 men, with a mean age of 60.7 ± 12.3 years (range, 25.0 to 81.0 years). The surgeons were blinded to group assignment preoperatively. Drainage samples were collected by one participant who weren’t blinded. Another two participants who evaluated the concentration and bioassays of the antibiotics were all blinded to group assignment.

### Surgical procedures

An institutional standard protocol of two-stage exchange arthroplasty was performed in all patients. Patients had their implanted components removed followed by thorough and radical debridement of the unvital bone and soft tissue. A minimum of three sets of cultures were obtained. After irrigation, a cement spacer loaded with two combined antibiotics was then implanted. All bone cement spacers were made intra-operatively by hand-mixing 4 g of vancomycin powder (VIANEX S.A., Athens, Greece) and 2 g of meropenem powder (Sumitomo Dainippon Pharma Co. Ltd., Osaka, Japan) per 40 g of methyl-methacrylate cement polymer (Heraeus Medical GmbH, Wehrheim/Ts., Germany). Then, liquid monomer was added and mixed for polymerization. The weight of the implanted cement spacer was recorded intra-operatively. A disposable, closed-suction drainage system (BDA-YS 400 ml; Branden, Shandong, China) was placed in all patients for at least five days. The drains were inserted under the fascia and connected to an evacuator via connector tube. A CSD was placed in all patients after the first-stage surgery to collect drainage for detection of antibiotic concentration. For the study group, the drainage tube remained clamped postoperatively during the study period, which was considered non-use of a CSD. For the control group, the drainage tube was clamped for only 2 h postoperatively and then remained open until removal of the drain. All patients received ceftriaxone (2 g IV) at the induction of anesthesia preoperatively. Intravenous (IV) linezolid (0.6 g IV q 12 h) and ceftriaxone (2 g IV q 24 h) were used in the first five days following spacer implantation. After the study period (5 days following spacer implantation), the antibiotic regimen was decided based on the intraoperative culture sensitivity reports and institutional guidelines.

After implantation of the cement spacer, 5-mL fresh aliquots of drainage were collected under sterile conditions every 24 h for the first five days, and the drainage container was changed at the time of drainage collection. After the given study period, the drain was removed if the daily drainage amount was less than 50 mL. Additionally, 10 mL of peripheral venous blood was collected during the first 24 h following implantation of the spacer. All samples were stored and frozen at − 80 °C for no more than three months. The reimplantation was performed after 2–4 weeks of antibiotic holiday and the soft tissue was free of local heat, erythema, swelling, and any infection-related symptoms.

### Determination of antibiotic concentrations

The concentrations of vancomycin and meropenem in the drainage were measured daily for 5 days by using high-performance liquid chromatography (HPLC) assay carried out on an Agilent 1260 Infinity chromatograph with a Thermo Hypersil C_18_ column (150 mm by 4.6 mm; 5 μm particle size). The standard calibration curve consisted of eleven different standard concentrations (0.625, 1.25, 2.5, 5, 10, 20, 40, 80, 160, 320, 640 and 1280 μg/mL). The mobile phase consisted of acetonitrile-10.53 mm ammonium acetate (composite ratio, 95/5, pH 4) for meropenem and monopotassium phosphate (25 mmol/L)-methanol (86/14, pH 2.4) for vancomycin. The flow-rate was 1.0 mL/min, and the detection wavelengths were 298 nm and 236 nm for meropenem and vancomycin, respectively. The injection volume was 20 μL, and the temperature of the column was 30 °C. The HPLC system had sensitivities of 0.5 μg/mL for vancomycin and 0.6 μg/mL for meropenem. The concentrations of antibiotics in the drainage samples were determined by comparison with the peak areas of standard curves prepared daily.

### Bioassay of antibiotic activity

The bioactivity of the drainage and peripheral venous blood were assessed using an agar disk diffusion bioassay, conducted as described by Hsu et al. [2] Discs containing 35 μL of joint fluid were placed on agar seeded with methicillin-sensitive *Staphylococcus aureus* (MSSA) (ATCC 25923), methicillin-resistant *Staphylococcus aureus* (MRSA) (ATCC 43300), and *E. coli* (ATCC 25922). Inhibitory activity of the disks was determined after 24-h incubation at 37 °C. The diameters of the inhibition zones were measured using a caliper. All samples were tested three times.

### Sample size calculation

A noninferiority test was conducted to determine the sample size. Prior study data have indicated 100% antibacterial activity of joint drainage in patients with a CSD in the first week following spacer implantation, [2, 12, 13] so we planned for a minimum expected antibacterial activity rate of 95%. We used a difference (delta value) of 20%, a power of 80%, and an alpha error of 0.05; a sample size of at least 14 for each group was determined. Totally, the present study included 32 patients with 160 drainage samples.

### Statistical analysis

Categorical variables were presented as frequencies and percentages and continuous variables as the means and standard deviation (M ± SD). The clinical characteristics between groups were compared with the use of the independent *t*-test or Mann-Whitney test for continuous variables and the chi-square test for categorical variables. Patients in the control group were further divided into two subgroups based on the median of total drainage volume (400 mL). Univariate linear regression analysis was used to examine the association between drainage volume (as both continuous and categorical variables) and antibiotic concentrations in the joint fluid. β-coefficient and 95% confidence intervals (CIs) were reported. A *p* value less than 0.05 was considered significant. All of the analyses were performed with the statistical software packages R (http://www.R-project.org, The R Foundation).

## Results

Patient demographics and organism profile at the time of resection arthroplasty are presented in Table 1, with comparable age, body mass index and ratios of gender. There were no significant differences in the amount of implanted cement (78.9 ± 12.8 vs. 78.3 ± 8.9 g) or antibiotics (11.8 ± 1.9 vs. 11.7 ± 1.3 g) between the study group and control group. Additionally, according to clinical and laboratory monitoring, no patient in this series presented any allergy, renal or hepatic dysfunction, or other adverse effects owing to antibiotic management.

**Table 1:** Patient characteristics

	Study group (*n* = 16)		Control group (n = 16)		P value
Female	10	(62.5%)	12	(75.0%)	0.704
Age (year)	61.9 ± 12.0		59.5 ± 12.9		0.485
BMI	26.1 ± 3.7		25.1 ± 3.0		0.346
Amount of implanted cement (g)	78.9 ± 12.8		78.3 ± 8.9		0.850
Amount of antibiotic (g)	11.8 ± 1.9		11.7 ± 1.3		0.985
Vancomycin (g)	7.9 ± 1.3		7.8 ± 0.9		
Meropenem (g)	3.9 ± 0.6		3.9 ± 0.4		
Organism culture					–
Coagulase negative Staphylococci	7 (43.8%)		6 (37.5%)		
*Staphylococcus aureus*	3 (18.8%)		4 (25.0%)		
Polymicrobial organism	1 (6.3%)		3 (18.8%)		
Gram-negative bacteria	2 (12.5%)		0		
Other organism	2 (12.5%)		0		
Culture negative	1 (6.3%)		3 (18.8%)		

### Antibiotic concentrations

**Fig. 2:**
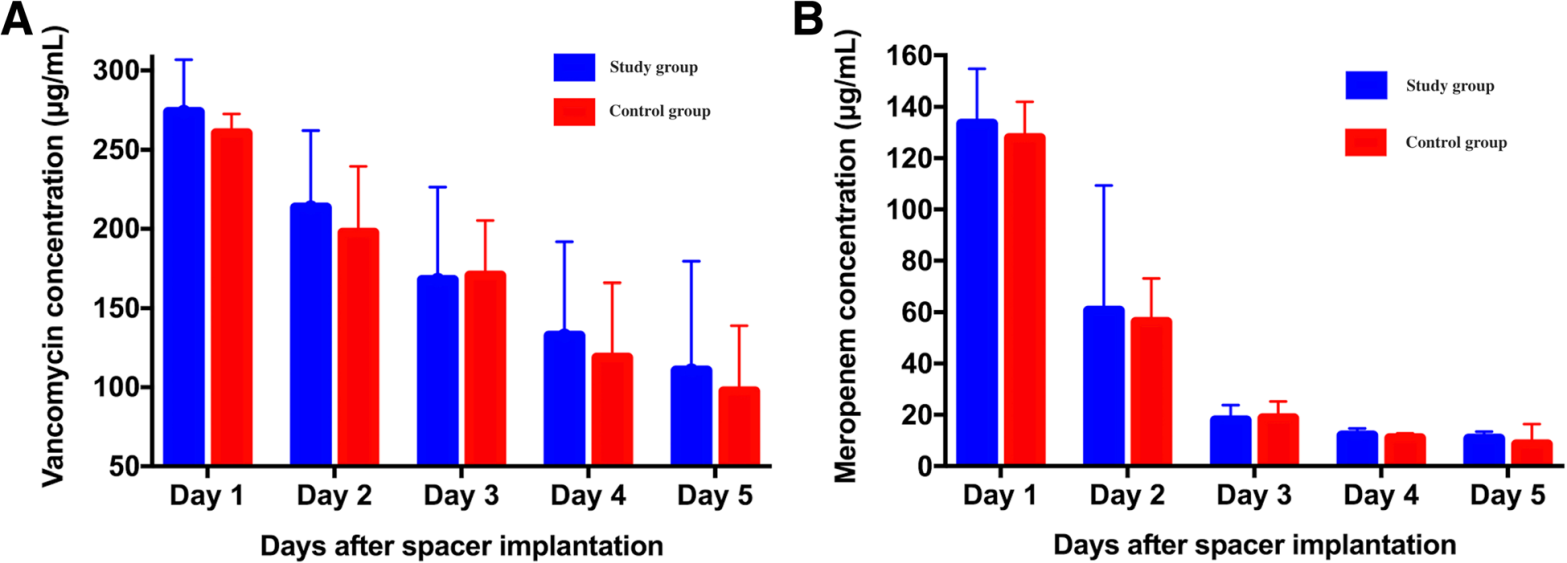
Antibiotic concentrations of vancomycin (A) and meropenem (B) in drainage samples in the first five days following cement spacer implantation

Both vancomycin and meropenem were burst released from the cement spacer during the first day of the elution assay in the drainage samples of all patients. The released rates of all the tested samples then gradually decreased by the next time points (Fig. 2A **and** Fig. 2B**;** Table 2). There was no significant difference in the decrease of vancomycin concentration between the two groups during the first five days following spacer implantation (study group vs. control group: 163.20 ± 77.05 vs. 162.39 ± 36.31; *p* = 0.917). The concentration of vancomycin in the non-CSD and CSD groups was 273.9 ± 32.9 and 260.5 ± 12.2 μg/mL, respectively, on the first day and decreased to 110.5 ± 69.1 and 97.5 ± 41.5 μg/mL, respectively, on the fifth day. The decrease of the meropenem concentration did not reach a significant difference between the two groups (study group vs. control group: 123.78 ± 21.04 vs. 117.27 ± 19.38; *P* = 0.548). The meropenem concentration of the study group and control group was 133.5 ± 21.2 and 127.9 ± 14.1 μg/mL, respectively, on the first day and decreased to 10.7 ± 2.7 and 8.5 ± 7.9 μg/mL, respectively, on the fifth day. The concentrations of vancomycin and meropenem in the serum were too low to be detected by our HPLC system in both groups.

**Table 2:** Postoperative antibiotic concentration

	Study group	Control group	*P*-value
Vancomycin concentration (M ± SD)			
Day 1	273.26 ± 31.93	260.85 ± 11.88	0.156
Day 2	214.39 ± 47.19	199.90 ± 41.68	0.365
Day 3	165.39 ± 57.49	173.02 ± 35.36	0.654
Day 4	131.92 ± 57.28	122.16 ± 47.73	0.604
Day 5	110.13 ± 66.78	98.52 ± 40.31	0.556
Change	163.20 ± 77.05	162.39 ± 36.31	0.917
Meropenem concentration (M ± SD)			
Day 1	134.04 ± 20.59	126.61 ± 14.56	0.248
Day 2	63.99 ± 27.28	56.84 ± 16.36	0.375
Day 3	18.09 ± 5.88	19.35 ± 6.86	0.581
Day 4	11.78 ± 2.79	10.93 ± 1.88	0.318
Day 5	10.27 ± 3.26	9.34 ± 8.40	0.682
Change	123.78 ± 21.04	117.27 ± 19.38	0.548

### Total drainage amounts and antibiotic concentrations (only in the CSD group)

Among patients in the CSD group, the linear regression analysis showed total drainage volumes (as a continuous variable) were not associated with antibiotic concentrations of the fifth day (for vancomycin concentration: β-coefficient, − 0.40; 95% CI, −-0.86 to 0.07, *p* = 0.116; for meropenem concentration: β-coefficient, 0.02; 95%CI, − 0.08 to 0.13, *p* = 0.673). When analyzed drainage volumes as a categorical variable (more than 400 mL or not), the results were consistent; there was no association between total drainage volumes and antibiotic concentrations of the fifth day (for Vancomycin concentration: β-coefficient, − 6.13; 95% CI, − 47.22 to 34.96, *p* = 0.774; for Meropenem concentration: β-coefficient, 4.83; 95%CI, − 3.38 to 13.03, *p* = 0.268) (Table 3).

**Table 3:** Association between drainage amount and antibiotic concentrations at day 5

	Vancomycin concentration		Meropenem concentration	
	β-coefficient (95% CI)	*p* value	β-coefficient (95% CI)	*p* value
Drain volume (ml)	−0.40 (−0.86, 0.07)	0.116	0.02 (−0.08, 0.13)	0.673
< 400 mL	Reference	–	Reference	–
≥ 400 mL	−6.13 (−47.22, 34.96)	0.774	4.83 (−3.38, 13.03)	0.268

### Bioassay of antibiotic activity

**Fig. 3:**
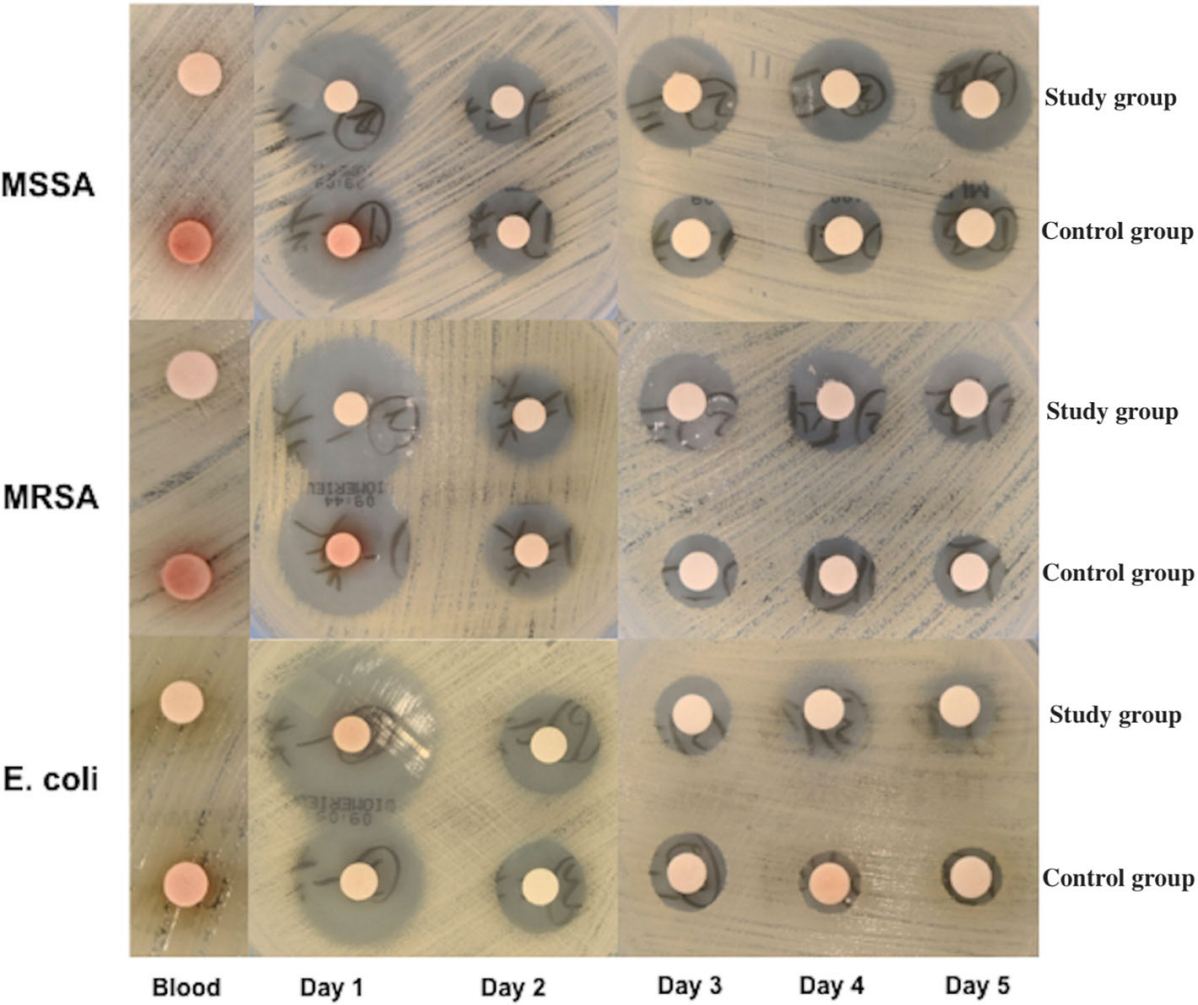
Antibacterial activities against test bacteria for the non-CSD and CSD groups

The drainage samples of all patients presented antibacterial activity against MSSA, MRSA and *E. coli* during the first five days following spacer implantation. Additionally, the inhibitory zone of all tested organisms gradually decreased during the study period (Fig. 3). In the disk diffusion assay, the peripheral venous blood sample of the first day exhibited limited antibacterial activity.

## Discussion

This study, the first of its kind of which we are aware, evaluated the influence of a CSD on antibiotic release from the antibiotic-loaded cement spacer in two-stage exchange arthroplasty. In this study, we presumed a CSD with clamped tube to be equivalent to no placement of CSD. The study group (non-CSD group) had similar vancomycin and meropenem concentrations compared to those of the control group (CSD group) up to 5 days following spacer implantation. This result showed that the use of CSD did not affect the local antibiotic concentrations from the antibiotic spacer. Moreover, the result suggested that higher drainage volume did not result in lower antibiotic concentrations detected in the drainage.

Prior studies have shown that local antibiotic concentrations could remain above their effective levels for a long period, both in vitro and in vivo, owing to the continuous antibiotic release from the antibiotic-loaded cement spacer [2, 14, 15]. In a study by Fink et al.,^13^ CSD was commonly used following spacer insertion. They inspected the tissue surrounding the spacer in the sixth week following cement spacer insertion. They found the concentration of antibiotics loaded in the cement spacer was still higher than the inhibitory concentration required for treating the pathogens that are responsible for most periprosthetic infections. In another study by Kelm et al.,^14^ a CSD was used in all patients for seven days following spacer implantation. The mean time of reimplantation was nine weeks, and they detected adequate antibiotic concentrations left over. The results also suggested that the antibiotic levels far exceeded therapeutic requirements against common microorganisms involved in PJI at the time of reimplantation.^14^ Similarly to our own results, these aforementioned studies have also potentially illustrated that the use of CSD may not be a detriment to the efficacy of antibiotic-loaded spacers. Furthermore, Anagnostakos et al. [16] conducted a systematic review and assumed that the fluid volume that eluted the antibiotics from the spacer might influence the wash-out capability, which was not always stated in the existing studies. In the present study, the result showed no association between drainage quantity and antibiotic concentrations. The rationale for these findings may be that the antibiotics removed by the drainage were only a fraction of the total eluted antibiotics, which was not enough to affect the antibiotic concentrations surrounding the spacer.

The types of antibiotics themselves mixed into bone cement have an impact on local concentrations and elution kinetics. The common antibiotics mixed into the bone cement include gentamicin, clindamycin, vancomycin, tobramycin, aztreonam, meropenem and ampicillin, [2, 14, 17] which should be thermostable and available in powder forms. The antibiotics in the bone cement should provide a broad spectrum of antimicrobial coverage and a long-term effectiveness. The combination of antibiotics in the bone cement could increase the porosity of the cement spacer and, hence, increase the release of antibiotics.^15^ Baleani et al. [18] showed the addition of meropenem to cement spacers increased the elution of vancomycin from the antibiotic-loaded bone cement. Hsu and colleagues conducted an in vitro study to compare six commonly used antibiotic combinations in bone cement, and they suggested that the combination of vancomycin and ceftazidime demonstrated a long-term antibacterial capacity.^2^ In our study, the combination of vancomycin and meropenem in the bone cement was utilized in accordance with our institutional infection control department, which explained that more than 90% of the organisms isolated from patients with PJI in our institutional were sensitive to one or both antibiotics.

It is critical to consider adverse effects owing to antibiotics used. Although vancomycin is one of the most commonly used antibiotics in cement spacers, the renal toxicity of vancomycin is the major concern. Several studies suggested that vancomycin in a cement spacer contributed to acute renal injury after implantation [19, 20]. However, Hsieh et al. and Springer et al. reported no systemic side effects attributed to the use of high doses of vancomycin in cement spacers [13, 21]. In the present study, antibiotics (vancomycin and meropenem) mixed into cement spacers were too low to be examined in the venous blood, which was similar to prior studies [2, 12, 22]. In addition, at the last follow-up, none of our study patients presented any findings of acute renal failure or side effects pertaining to antibiotic applications.

When interpreting our findings, several limitations should be considered. First, given the ethical concerns and risk of organism contamination, we only kept the drains in patients for five days. Additionally, all patients in the non-CSD group opened their CSDs after the study period (5 days) to drain out residual joint fluids due to the requirement by our ethics committee. Therefore, this study was unable to compare postoperative wound complications between groups. Second, the concentration of meropenem decreased dramatically compared to 4antibiotic concentrations would remain higher than the minimum inhibitory concentration over the period of the spacer. Third, we only tested the antibiotic bioactivity of the drainage samples against MSSA, MRSA and *E.coli* in the experiment. Other common PJI organisms were not evaluated. Fourth, for detecting antibiotic concentration, we had to use CSD in the study group to collect joint fluid postoperatively. However, the CSD tube kept clamped unless only 5-mL drainage were collected every 24 h for the first five days in the study group. Fifth, the sample size may have been inadequate for conducting subgroup statistical analyses, and the possibility of a type-II error existed. Last, we did not consider the impact of intravenous antibiotics on the release of antibiotics in the spacer. However, the intravenous antibiotic regimen (linezolid and ceftriaxone) was different from the local antibiotic regimen (vancomycin and meropenem) to avoid detection bias.

## Conclusions

In conclusion, this randomized, controlled trial suggested that the use of a closed-suction drainage does not reduce the effectiveness of an antibiotic-loaded spacer in two-stage exchange arthroplasty. Further studies may be necessary to evaluate the outcome of treatment and postoperative complications of two-stage exchange arthroplasty with or without the use of closed-suction drainage.

## Data Availability

Data are available on request from the authors.

## Abbreviations

ACS: Antibiotic-loaded cement spacer
CIs: Confidence intervals
CSD: Closed-suction drain
HPLC: High-performance liquid chromatography
ICM: International Consensus Meeting
MSIS: Musculoskeletal Infection Society
MSRA: Methicillin-resistant *Staphylococcus aureus*
MSSA: Methicillin-sensitive *Staphylococcus aureus*
PJI: Periprosthetic joint infection
THA: Total hip arthroplasty
TJA: Total joint arthroplasty
